# Acaricidal Toxicity of Four Essential Oils, Their Predominant Constituents, Their Mixtures against *Varroa* Mite, and Their Selectivity to Honey Bees (*Apis cerana* and *A. mellifera*)

**DOI:** 10.3390/insects14090735

**Published:** 2023-08-30

**Authors:** Tekalign Begna, Delgermaa Ulziibayar, Daniel Bisrat, Chuleui Jung

**Affiliations:** 1 Department of Plant Medicals, Andong National University, Andong 36729, Republic of Korea; tekalign12@gmail.com; 2 Department of Environmental Technology, School of Tourism and Land Management, Mongolian National University, Ulanbator P.O. Box -46A/523, Mongolia; u.deegii7@gmail.com; 3 Department of Pharmaceutical Chemistry and Pharmacognosy, School of Pharmacy, College of Health Sciences, Addis Ababa University, Addis Ababa P.O. Box 1176, Ethiopia; danielbisrat@gmail.com; 4 Agriculture Science and Technology Research Institute, Andong National University, Andong 36729, Republic of Korea

**Keywords:** complete exposure, mixture, selectivity ratio, thymol, toxicity, *Trachyspermum ammi*, *Varroa destructor*

## Abstract

**Simple Summary:**

Honey bees (*Apis mellifera*) that play vital roles in pollination and ecosystem maintenance, face severe threats from the ectoparasite, *Varroa destructor.* Existing control techniques, including mechanical, chemical, and organic, have had adverse effects on honey bees. Therefore, finding an easy, effective, affordable, and safe method is crucial. Essential oils (EOs) and their major components emerge as potential candidates due to their higher efficiency, biodegradability, and selectivity. However, evaluating composition variability, as well as their efficiency and safety in honey bee species, is essential. In this study, we assessed the efficiency of essential oil and their components against *Varroa* mites while studying the safety for honey bees. *Eucalyptus globulus*, *Rosemary officinalis*, *Trachyspermum ammi* (Ethiopian and Indian varieties), alongside their major components and a 1:1 mixture, were assessed for their acaricidal activity. All the samples exhibited acaricidal activity, with *T. ammi*, thymol, and the 1:1 mixture of thymol and carvacrol showing the highest efficiency against *V. destructor*. Importantly, the EOs and their major components showed selectivity and did not affect the honey bees’ learning and memory. In conclusion, our findings highlight the potential of *T. ammi* and the 1:1 mixture of thymol and carvacrol as candidates for *Varroa* control, suggesting further study at the colony level.

**Abstract:**

The honey bee (*Apis mellifera*) faces a significant threat from *Varroa destructor*, causing the losses of millions of colonies worldwide. While synthetic acaricides are widely used to control *Varroa* infestations, excessive application has led to resistant strains and poses side effects on the host. Consequently, there is an urgent need for a new acaricide that is both effective and affordable, yet safe to use on bees. One potential source of these acaricides is essential oils (EOs) and their constituents. This study evaluated the acaricidal properties of four essential oils (*Eucalyptus globulus*, *Rosemary officinalis*, *Trachyspermum ammi* (Ethiopian and Indian varieties), their constituents and mixture of constituents against *V. destructor* through the complete exposure method. Our finding showed that a 1:1 mixture of thymol and carvacrol (4 h-LC_50_ = 42 μg/mL), thymol (4 h-LC_50_ = 71 μg/mL), and *T. ammi* oil (4 h-LC_50_ = 81–98 μg/mL) were the most toxic test samples against *V. destructor*. Honey bee behavior and selectivity were also assessed with one additional EO *Thymus schimperi*, indicating that *T. schimperi*, *T. ammi*, and their components were selective and did not affect the learning and memory of bees. In conclusion, the thymol and carvacrol (1:1) mixture was shown to be a promising replacement for synthetic acaricides, being three times more toxic than a commercial acaricide, fluvalinate (4 h-LC_50_ = 143 μg/mL).

## 1. Introduction

Honey bees, primarily *A. mellifera* Linnaeus remain one of the most economically valuable pollinators of crop and wild plants worldwide [1]. In the absence of pollinators, over 90% crop yield decline was reported, particularly in certain fruit, seed, and nut crops [2]. Because of their economic importance, the Western honey bees, *A. mellifera*, native to Europe, Africa, and the Middle East have been repeatedly introduced in almost all regions of the world [3]. Following its introduction, *A. mellifera* came into contact with a broad range of parasites and pathogens infecting Asian Honey bees, *Apis cerana* Fabricius [4]. Among these, *Varroa destructor* Anderson and Trueman, mites initially infested *A. mellifera* between the 1940s and 1950s [5].

In recent decades, the beekeeping industry has been facing a serious global threat from the *V. destructor* mite [6,7], which is known to cause significant losses of bee colonies during the winter season [8]. This infestation has been linked to reduced honey bee colonies in various parts of the world, including the United States (30%), Europe (up to 53%), the Middle East (10–85%), and Japan (25%) as the United Nations Environment Programme (UNEP) emerging issues reported [9]. It was also reported that *V. destructor* has been the most significant threat to honey bees colonies in Korea [10], the United States [11], and New Zealand [12].

While conventional acaricides have been used to control the *V. destructor* mite, their overuse has resulted in mite resistance and the accumulation of high levels of miticides and their metabolites in honey bee colonies [13,14,15]. These chemicals can cause side effects [16], such as disruption of brood development and effects on learning and memory, longevity, colony strength [17], as well as queen and drone reproduction [17,18]. In addition, the use of conventional acaricides can affect the hygiene and the foraging behavior of bees [19], increasing the risk of colony collapse when combined with additional stressors [17]. Therefore, it has become crucial to investigate natural alternatives such as bio-pesticides, which are considered safer and more environmental friendly [20].

Essential oils (EOs) from various plant parts have diverse applications in industries, such as for perfume, food, cosmetics, pharmaceuticals, and beverages [21,22] and have been studied for their potential pharmacological and cosmetic utility due to their antioxidant, antimicrobial, anticancer, anti-inflammatory, anti-aging, and melanogenesis-inhibiting capabilities [21].

Since the 1990s, researchers have been exploring the potential of EOs and their components as alternative methods for controlling *V. destructor* in the laboratory and field [23,24,25,26,27,28,29,30,31]. Several studies have indicated that EOs demonstrate effectiveness in mite control while being safe to honey bees [32,33]. Additionally, it has been shown that the main components of essential oils are effective in controlling *Varroa* mites [28,34]. Some reports suggest that EOs or their major components might be more toxic to honey bees. Martinez et al. [35] reported that the essential oil of *Cymbopogon nardus* (L.) Rendle from Argentina showed promise as a candidate for controlling *V. destructor* due to its low toxicity against bees. However, the study also revealed that its major component, citronellal, exhibited a higher level of toxicity to honey bees.

In this study, we characterized the chemical composition of EO extracts from *Eucalyptus globulus Labill.*, *Rosmarinus officinalis* L. and *Trachyspermum ammi* (L.) Sprague (both Ethiopian and Indian varieties). We assessed their toxicity against the honey bee mite, *V. destructor*, and also evaluated the toxicity of their main constituents against *Varroa* mites. Additionally, we examined the toxicity of more toxic EOs, along with major components and *Thymus schimperi* Ronniger EO, including its main component carvacrol (reported for its toxicity to *Varroa* mites, Bisrat et al. [28]), against two honey bee species (*A. cerana* and *A. mellifera*) for selectivity testing. Moreover, we investigated the effects of *T. ammi*, *T. schimperi*, and their major components thymol, carvacrol, and γ-terpinene on learning and memory in *A. mellifera*.

## 2. Materials and Methods

### 2.1. Materials 

#### 2.1.1. Plant Materials

The plant materials used in the study included fresh leaves of *E. globulus* and *R. officinalis* acquired from southern and central Ethiopian regions, respectively. *T. ammi* seeds were obtained from two different sources: the Ethiopian variety was purchased from a marketplace in Holeta, Ethiopia while the Indian variety, was acquired from Raja Foods (batch number 32919) through an online market. All plant materials collected from Ethiopia were authenticated at the National Herbarium, Department of Biology, Addis Ababa University, Ethiopia.

#### 2.1.2. Chemicals

Thymol (purity = 98.5%, CAS-No. 89-83-8), Deajung reagents chemicals, Siheung, Republic of Korea, carvacrol (purity = 97%, CAS-No. 99-85-4), Sigma Aldrich, Bengaluru, India, p-cymene (purity = 99%, CAS-No. 99-87-6), Sigma Aldrich, Massachusetts, USA, 1,8-cineole (purity > 95%, CAS-No. 470-82-6; Sigma Aldrich, Sydney, Australia, γ-terpinene (purity = 97%, CAS-No. 99-85-4), Sigma Aldrich, Buchs, Switzerland, 1-nonanol (purity > 98%; CAS No. 143–08-8), Sigma Aldrich, Gillingham, UK, Fluvalinate (purity = 95%, CAS-No. 102851-06-9), Sigma Aldrich, Buchs, Switzerland, amitraz (purity > 98%, CAS No 33089-61-1) Sigma-Aldrich, MA, USA) and acetone (purity = 99.5%; CAS No. 67-64-1; Daejung reagents chemicals, Siheung, Republic of Korea) were purchased and EOs were extracted and prepared as described below.

#### 2.1.3. GC-MS Instrument

A gas chromatographic-mass spectrometric (GC-MS) analysis of the EO was performed on an Agilent 7890B Gas Chromatography system (Agilent Technologies, Wilmington, NC, USA), coupled to an Agilent 5977A Mass Spectrometer Detector system (Agilent Technologies, Wilmington, USA).

#### 2.1.4. Varroa Mite Collection

Female adult *V. destructor* were collected from *A. mellifera* colonies in the experimental apiary of the Andong National University, Andong, Republic of Korea using colonies that had not been treated with miticides for at least one year. The *V. destructors* were separated from the bees by shaking them in a jar with two table spoons of powdered sugar and collecting them in a sieve. Five active female mites were selected under a microscope and transferred to a 20 mL vial pre-treated with a predetermined concentration of treatments.

#### 2.1.5. Honey Bees

Workers of mixed age from heathy queenright colonies of *A. cerana* and *A. mellifera* were collected from the experimental apiary listed above on a sunny and warm day using the method described by [36]. Bees were collected from frames by brushing them into a 120 × 80 mm insect breeding dish (SPL-TDS-ISBDJ, Pocheon, Republic of Korea) and transported to the laboratory for toxicity bioassay. The collected bees were maintained at 25 ± 2 °C, 60 ± 10% RH and provided with a 50% sucrose solution until the start of the bioassay.

For learning and memory bioassay, returning pollen foragers from three healthy queenright *A. mellifera* colonies were collected individually at the entrance with 20 mL vials from the same listed apiary. The vials were then taken to the lab and fed to satiety with a 50% sugar solution and left in the dark at room temperature, 60% RH, until the analysis began [37]. Before being inserted into appropriately cut 1 mL plastic pipette tips that permitted free movement of their antennas and mouthparts, the bees were refrigerated for 3–5 min, following the description provided previously [38].

### 2.2. Methods

#### 2.2.1. Extraction of Essential Oils from Plant Species

Four EO-bearing plants (*T. ammi* Indian and Ethiopian varieties; 250 g seed, each; *E. globulus* 500 g, leaf; *R. officinalis* 500 g, leaf) were subjected to hydrodistillation using a Clevenger-type apparatus for 3 h. The distillates dried on anhydrous sodium sulphate produced oils and were stored in sealed glass vials at 4 °C prior to analysis. The EO yield was expressed in *v*/*w*% as a function of the weight of fresh plant material. Furthermore, in addition to the four essential oils that were extracted and tested against *Varroa* mites, we obtained *T. schimperi* EO and its major components from a previous study [28]. This particular essential oil has been reported as a potential candidate for controlling *Varroa* mites. Thus, our objective was to examine its toxicity effects on two honey bee species.

#### 2.2.2. GC-MS Analysis Conditions

An HP5-MS capillary column (a non-polar column; 30 m × 0.25 mm and 0.25 µm film thickness, Agilent Technologies, Wilmington, USA) was used to separate and analyze individual components. Then, 1 µL of a diluted sample (1/100; *v*/*v*, EO in acetone) was injected in split mode with a split ratio of 1:20. The gas chromatographic conditions were carrier gas helium (1.0 mL/min), an initial oven temperature of 40 °C for 3 min isothermal, 40 to 150 °C at a rate of 6 °C/min, and 150 to 320 °C at a rate of 10 °C/min, then held for 3 min. The injector temperature was set to 270 °C. Mass spectra were scanned in the range 40 to 500 amu with EI mode (70 eV) in full scan mode. The percentage composition of the EO was calculated using the peak normalization method. The EO constituents were identified by comparing their retention indices (RI), mass spectra with NIST (National Institute of Standards and Technology), Adams library spectra [39], Wiley 7 n.1 mass computer library, and general characteristics in the published literature [40].

#### 2.2.3. Toxicity of Essential Oils and Their Major Constituents against *V. destructor*

In this study, we employed the complete exposure method to assess the toxicity of essential oils (EOs) and their major constituents against *V. destructor*. Our evaluation specifically focused on the acaricidal toxicity of EOs and their major components, with a particular emphasis on the highly efficient EOs *T. ammi* and *T. schimperi* [28]. Additionally, we investigated the acaricidal toxicity of mixtures containing thymol:carvacrol (1:1), thymol:γ-terpinene (1:1), and thymol:carvacrol:γ-terpinene (1:1:1) using a methodology described elsewhere [41]. For each test sample, we prepared five concentrations by serially diluting them in acetone from a stock solution of 50 mg/mL. The concentration ranges were 625 to 10,000 µg/mL for *E. globulus* and *R. officinalis*, 15.75–250 µg/mL for thymol, 31.25 to 500 µg/mL for *T. ammi*, as well as all three mixtures (thymol:carvacrol, thymol:γ-terpinene, and thymol:carvacrol:γ-terpinene). Additionally, γ-terpinene concentrations ranged from 156.25 to 2500 µg/mL, as determined from a preliminary study. Fluvalinate and acetone served as the positive and solvent controls, respectively. To begin the experiment, we introduced 1 mL of each solution into a 20 mL glass scintillation vial using a micropipette [42]. The vials were then rolled to distribute the solution on the inner walls and subsequently pumped with nitrogen gas to evaporate the acetone. The vials were adequately sealed until the next step. Then, we introduced five active female *V. destructor* mites into each pretreated vial using a fine paintbrush and incubated them at a temperature of 28 °C and 70% relative humidity. The entire experiment was conducted in triplicate, and each individual test sample consisted of 75 female *V. destructor*. After four hours of treatment, we recorded the number of dead mites under a microscope. Mites were considered dead if they displayed no movement when touched with a fine-tipped brush under the microscope.

#### 2.2.4. Toxicity of *T. ammi* and *T. schimperi* on Honey Bees

The effects of *T. ammi*, *T. schimperi*, and their major components were evaluated on honey bees with some modifications to the method by da Silva et al. [43]. Honey bees were anesthetized using CO_2_ for proper handling during the experiment [44]. We established five concentrations for each treatment by serial dilution with the same stock solution used in mite toxicity bioassay. The concentration ranges were 31.25 to 500 µg/mL for carvacrol and thymol, 62.5 to 1000 µg/mL for *T. ammi* and *T. schimperi*, and 625.5 to 10,000 µg/mL for γ-terpinene, based on preliminary testing. The honey bees were exposed to these concentrations in three replications, with each test sample consisting of 150 *A. cerana* and 180 *A. mellifera* and incubated at a temperature of 28 °C and 70% relative humidity.

##### Surface Treatment Bioassay

Surface treatment bioassays were conducted on *T. ammi*, *T. schimperi*, and their major components, which include thymol, carvacrol, and γ-terpinene. Acetone and fluvalinate were used as the solvent and positive controls, respectively. For each individual test concentration mentioned above, 1 mL was applied to a filter paper (90 mm, Filter paper qualitative, Advantec^®^, circle, Toyo Roshi Kaisha Ltd., Tokyo, Japan), which was left to dry within the testing insect rearing cages for an hour. Ten *A. cerana* and twelve *A. mellifera* were placed in separate cages lined with the treated filter paper, and the experiment was repeated three times. Sugar solution and water were provided through pierced holes sealed with paraffin. Bee mortality was recorded after 4, 8, 12, 24, 48, and 72 h. Honey bees were counted as dead when complete immobility was observed following a gentle examination under ambient light outside the incubator

##### Topical Application Bioassay

The high susceptibility of *A. mellifera* to *V. destructor*, which potentially utilizes effective EOs and their components [29], drove us in the present study to further evaluation of more effective EOs by topical exposure bioassay. In order to achieve this objective, we conducted a study to assess the toxicity of *T. ammi* and *T. schimperi*, as well as their major components, on *A. mellifera*. A total of 5 µL of *T. ammi*, *T. schimperi*, and carvacrol at concentrations of (125, 250, 500, 1000, and 2000 µg/mL), thymol at concentrations of (31.25, 62.5, 125, 250, and 500 µg/mL), and γ-terpinene at concentrations of (1250, 2500, 5000, 10,000, and 20,000 µg/mL) and Amitraz at concentrations of (38, 76, 152, 304, and 602 µg/mL) were administered on the thorax of twelve *A. mellifera*. The experiment was replicated three times. Additionally, acetone was used as a solvent control, and a negative control group, which received no treatment, was also included in the study. The bees were then transferred to insect rearing cages (phytohealth (103 × 78.6 mm) and (cap), clear polypropylene, SPL Life Science.co. Ltd., Pocheon, Republic of Korea) incubated at a temperature of 28 °C and 70% relative humidity, while bee mortality was recorded at 4, 24, 48, and 72 h after treatment. Honey bees were counted as dead when complete immobility was observed after a gentle probe with a fine brush. During the experiment, bees were provided with water and 50% sugar solution.

#### 2.2.5. Learning and Memory Bioassay

Learning and memory tests were conducted following the method described by [38], with pollen forager bees exposed to sub-lethal doses (LD_10_ and LD_20_) of *T. schimperi* (0.1 and 0.2 µg/bee), *T. ammi* oils (0.4 and 1.4 µg/bee) and their main constituents, thymol (0.5, 1 µg/bee), carvacrol (1.3, 2.6 µg/bee), and γ-terpinene (2.7, 9 µg/bee) before 4 h of the first conditioning trial by topical exposure. Furthermore, honey bees were subjected to amitraz (0.4, 1 µg/bee) exposure as a positive control, while an untreated acetone (0 µg/bee) group was used as the solvent control. Bees were gently harnessed into plastic pipette tips (1 mL) individually with only antennae and mouthparts being free to move. Prior to the analysis, the harnessed bees were fed a 50% sugar solution, then left in a dark place at room temperature and 60% RH. During learning trials, each harnessed bee was placed on the rack for 25 s. A conditioned stimulus (CS), containing 5 μL aliquot of 1-nonanol, was applied to a piece of filter paper (10 mm × 30 mm) placed inside a 20 mL syringe (Korean Vaccine Co., Ltd. Ansan, Republic of Korea) to onset the odor to each harnessed bee antennae for 4 s during each trial. During the CS presentation, the antenna of the harnessed bee was initially stimulated with a toothpick soaked in a 50% sugar solution. Subsequently, if the bee extended its proboscis, the bee was allowed to lick the unconditioned stimulus (US) for 3 s, overlapping with the CS presentation by 1 s. After the presentation of the US, the bee was left on the rack for an additional 25 s before being removed and replaced by the next bee. Each bee underwent six trials, with a ten-minute inter-trial interval (ITI) for proboscis extension reflex (PER) conditioning.

Memory was tested 28 h after the exposure to treatments, with CS presented for 4 s to each harnessed forager’s antenna in which US was not given, after each bee was placed at the conditioning site for 25 s.

#### 2.2.6. Statistical Analysis

For individual test samples, regression lines, 4 h-LC_50_ values, χ^2^ and 95% confidence limits were calculated from toxicity test responses of EOs and their major constituents to adult female *V. destructor* and honey bees using the Probit analysis. The reason for selecting this analysis was that toxicity could be determined based on a binomial response of either mites/honey bees being alive or dead. The statistical significance of mortality differences between EOs and their main components was examined using the chi-square independence test.

The selectivity ratio (SR) is the indication of chemical safety limits calculated by dividing the LC_50_ of honey bees by the LC_50_ *V. destructor*. When the SR ≤ 1, the chemical is non-selective toward the host. However, when SR > 1, the chemical becomes selective or harmless to the host [45].

The additive index (AI) used for measuring the combined toxicity of the major components was conducted as previously outlined in [46].

$$S=\frac{LC_{50}\;\mathrm{of}\;\mathrm{component}\;A\;\mathrm{in}\;\mathrm{mixture}}{LC_{50}\;\mathrm{of}\;\mathrm{component}\;A}+\frac{LC_{50}\;\mathrm{of}\;\mathrm{component}\;B\;\mathrm{in}\;\mathrm{mixture}}{LC_{50}\;\mathrm{of}\;\mathrm{component}\;B}$$

where: S is sum of the toxicity of component A and B; then AI is calculated as below:

$$\mathrm{AI}=\frac{1}{S}-1\;\mathrm{for}\;S<1,\;\mathrm{and}\;\mathrm{AI}=1-S\;for\;S=1.$$

If the value of the AI is less than or equal to −0.2, the combined effect is antagonistic, while it is additive if the AI falls between −0.2 and 0.25, and synergistic if the AI is greater than 0.25. Moreover, synergistic effect increases as the AI value increases.

Learning and memory responses were recorded as binary values, with 0 representing no response and 1 representing a proboscis extension response. The percentage of proboscis extension response (% PER) in learning and memory is calculated as the number of bees showing PER to the conditioned odor with respect to the total number of bees assayed [38]. To analyze the PER, a binary logistic regression was performed using a generalized linear model (GLM). The predictors in the analysis of PER responses were treatments, the number of learning trials, and the time of memory test. To determine the influence of fixed effects, *p*-values were obtained by analyzing the deviation table using Wald chi-square tests. All experimental analyses were conducted using SPSS version 16, SPSS Inc., Chicago, IL, 2007.

## 3. Results

### 3.1. Chemical Composition of Essential Oils

The percentage yields, color, and odor of the EOs obtained from hydrodistillation of each of the four plants are summarized in Table 1. *T. ammi* (Indian variety) exhibited the highest yield (4.1%), followed by *T. ammi* (Ethiopian variety) (3.7%), while the lowest yield was recorded for *R. officinalis* (0.83%).

Table 2 presents the major chemical compositions of each of the four EOs as identified by GC-MS analysis. The EOs of *E. globulus*, *R. officinalis*, *T. ammi* (Ethiopia), and *T. ammi* (India) consisted of seventeen, twenty, twenty-two, and seventeen compounds, respectively. The oils from these plants were predominately characterized by a high level of monoterpenes, namely 1, 8-cineole, carvacrol, thymol, γ-terpinene, p-cymene, α-pinene, and camphor but with varying composition percentages as outlined in Table 2. Additionally, Table S1 provides a detailed composition of the main components comprising EOs.

### 3.2. Acaricidal Activities of EOs and Their Main Components against *V. destructor*

The 4 h-LC_50_ values of the main components of each of the four EOs and some selected blends against *V. destructor* are presented in Table 3. Although all four EOs displayed acaricidal activity, the degree of their activity varied. On a 4 h post-treatment assay, a positive linear relationship was observed between the probit-transformed mortality values and the log-transformed concentration of the individual tested EOs (Figure S1). Consequently, the 4 h-LC_50_ values for each individual oil were determined from the probit mortality-log dose graph. Considering their 4 h-LC_50_ values, *T. ammi* EOs (Indian variety; 4 h-LC_50_ = 81 µg/mL and Ethiopian variety; 4 h-LC_50_ = 98 µg/mL) showed the highest toxicity to *Varroa* mites. No significant difference was observed in the mortality rate of *V. destructor* exposed to *T. ammi* EOs from both Ethiopia and India (χ^2^ = 0.111, df = 1, *p* = 0.739), and all follow-up studies were hence carried out on the Ethiopian variety.

During the course of the toxicity assay, *Varroa* mites exhibited signs of toxicity upon exposure to the *T. ammi* EOs, including restlessness and fast walking followed by slowing down. However, the lowest toxicities against *V. destructor* were observed for EOs from *E. globulus* (4 h LC_50_ = 4341 µg/mL) and *R. officinalis* (4 h-LC_50_ = 2577 µg/mL). Owing to the high toxicity of *T. ammi*, its main constituents (thymol, γ-terpinene, and p-cymene) were further evaluated. Thymol exhibited high acaricidal toxicity with 4-h LC_50_ values of 71.0 µg/mL. However, γ-terpinene (4 h-LC_50_ = 1339 µg/mL) and 1,8-cinoele (4 h-LC_50_ = 13,647 µg/mL) had low toxicity, and p-cymene did not cause any mite mortality even 24 h after treatment (Table 3).

The interaction among the three major constituents that exhibited acaricidal activity was then studied. Thymol and carvacrol in a binary 1:1 ratio showed a synergetic interaction with an AI of 1.0, resulting in increased toxicity against the *Varroa* mites (4 h-LC_50_ = 42.0 µg/mL or 2.0 mg/L air volume). However, antagonistic interactions were found in both the binary combination of thymol and γ-terpinene and the ternary mixture of thymol, carvacrol, and γ-terpinene, with AI values of −0.53 and −0.13, respectively.

### 3.3. Honey Bee Toxicity

Several experiments were conducted to assess the safety of *T. ammi* and *T. schimperi* EOs on two bee species (*A. cerana* and *A. mellifera*) via surface treatment bioassay, owing to their potent toxicity to mites. Positive linear relationships were observed between the probit-transformed mortality values and the log-transformed concentration of the oil and major components of *T. ammi* and *T. schimperi* for both bee species (Figures S2 and S3). *T. ammi* showed toxicity on *A. cerana* at a high concentration (1000 µg/mL: χ^2^ = 15.556, df = 1, *p* < 0.001) and 500 µg/mL: χ^2^ = 5.455, df = 1, *p* = 0.02) compared to the negative control, while no toxicity was noted at lower concentrations (*p* > 0.05). *T. ammi* oil was found to be 9× and 35× less toxic to *A. cerana* and *A. mellifera*, respectively, when compared with fluvalinate. Similarly, *T. schimperi* EO was shown to be 24× and 74× less toxic to *A. cerana* and *A. mellifera*, respectively, when compared with fluvalinate (Table S2).

Additionally, a topical bioassay was conducted to assess the toxicity of these EOs to *A. mellifera*, considering their high susceptibility to *V. destructor*. We observed positive linear correlations between the probit-transformed mortality rates and the log-transformed doses of *T. ammi*, *T. schimperi* oils, and their major components for *A. mellifera* (Figure S4). As summarized in Table 4, *T. ammi* EO, *T. schimperi* EO, and their major components showed low to moderate toxicity towards *A. mellifera*, with a 4 h-LD_50_ ranging from 4.6 to 86.5 µg/bee. Furthermore, *T. ammi* and γ-terpinene demonstrated lower toxicity towards *A. mellifera*, and their mortality rates were not significantly different from the solvent control (χ^2^ = 9.36, df = 5, *p* = 0.154) and γ-terpinene (χ^2^ = 9.30, df = 5, *p* = 0.096). However, *T. schimperi* (χ^2^ = 18.00, df = 5, *p* = 0.012), thymol (χ^2^ = 7.200, df = 5, and *p* = 0.006), and carvacrol (χ^2^ = 18.00, df = 5, *p* = 0.003) caused significantly higher mortality when compared to the solvent control, respectively).

The essential oils (EOs) and major components exhibited selective toxicity against *V. destructor* (SR > 1). *T. schimperi* and *T. ammi* displayed approximately 91- and 48-times higher toxicity to *Varroa* mites than to *A. mellifera*, respectively. Similarly, *T. schimperi* and *T. ammi* showed approximately 27- and 9-times greater toxicity to *Varroa* mites compared to *A. cerana*, respectively (Table 5).

### 3.4. Learning and Memory Bioassay

**Figure 1:**
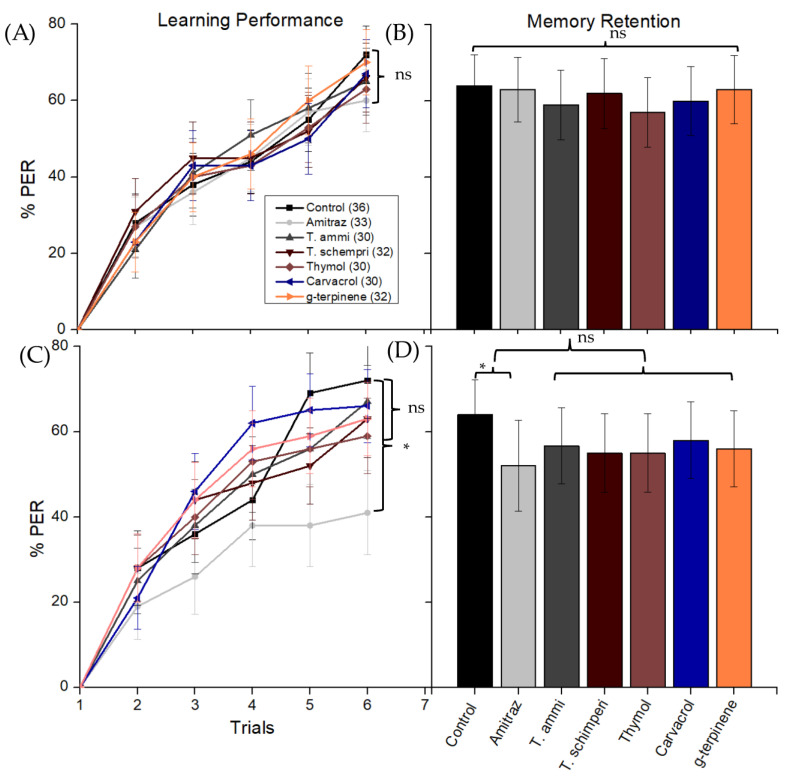
The learning performance (**A**,**C**) and memory retention (**B**,**D**) of honey bees conditioned with 1-nonanol assessed following 4 h of topical exposure to various substances. The substances tested, along with their LD_10_ values (0, 0.4, 0.4, 0.1, 0.5, 1.3, and 2.7 µg/bee, respectively) and LD_20_ values (0, 1, 1.4, 0.2, 1, 2.6, and 9.0 µg/bee, respectively), were control (n = 36), amitraz (n = 33), *T. ammi* (30), *T. schempri* (n = 32), thymol (n = 30), carvacrol (n = 30), and γ-terpinene (n = 32). The significant differences between the experimental groups are indicated (*) and ns indicated not significant between experimental groups.

Figure 1 depicts that the olfactory learning of *T. schimperi* and its main components, thymol and carvacrol, at LD_10_ and LD_20_ had no significant effect on proboscis extension response (PER) success, 4 h post treatments. Comparing control bees (0 µg/bee) to bees exposed to *T. schimperi* (χ^2^ = 0.522, df = 2, *p* = 0.47), thymol (χ^2^ = 0.000, df = 1, *p* = 0.993) and carvacrol (χ^2^ = 0.311, df = 1, *p* = 0.577) showed no significant differences in PER responses respectively) (Figure 1A,C). Similarly, exposure of foragers to *T. ammi* and its main components (thymol, γ-terpinene) at LD_10_ and LD_20_ had no significant effect on PER response 4 h after exposure (χ^2^ = 0.550, df = 4, *p* = 0.968) (Figure 1). Pairwise comparisons revealed no significant differences between the control and *T. ammi*, responses (χ^2^ = 0.000, df = 1, *p* = 0.998); thymol (χ^2^ = 0.025, df = 1, *p* = 0.875); γ-terpinene (χ^2^ = 0.009, df = 1, *p* = 0.668), in PER respectively) (Figure 1A,C). Moreover, the PER learning responses from LD_10_ and LD_20_ doses for each treatment were also insignificant (χ^2^ = 0.194, df = 1, *p* = 0.659) except for the amitraz. The sub-lethal dose of EOs and their main components showed a significance effect on learning performance in amitraz at higher dose (1 µg/bee) (*p* < 0.05) (Figure 1C), and a significant difference was noted between the number of trials (trial 1 and 6) (χ^2^ = 99.57, df = 4, *p* < 0.001).

The memory retention of foragers was found to be unaffected by *T. schimperi* and its main components, thymol and carvacrol (χ^2^ = 2.208, df = 4, *p* = 0.698). Similarly, *T. ammi* and its main components, thymol and γ-terpinene, did not significantly impact memory retention (χ^2^ = 1.822, df = 4, *p* = 0.768) (Figure 1B,D).

## 4. Discussion

In the present study, we found that *Varroa* mites exhibited signs of toxicity upon exposure to the *T. ammi* and *T. schimperi* EOs, including restlessness and fast walking followed by slowing down during the toxicity assay. Most importantly, our results indicated that EOs obtained from two plants, *T. ammi* (4 h-LC_50_ = 81–98 µg/mL) and *T. schimperi* (4 h-LC_50_ = 109 µg/mL) Bisrat et al. [28] and their major constituents (thymol; 4 h-LC_50_ = 71.0 µg/mL; carvacrol 4 h-LC_50_ = 106.0 µg/mL) Bisrat et al. [28] were without any negative effects on honey bee survival or behavior. Moreover, the study found that a mixture of major components (thymol:carvacrol; 1:1) had a synergistic effect on the mites.

The chemical compositions of EOs are influenced by various factors such as plant part, extraction method, and geographical location [47] resulting in different biological activity. *T. ammi* oil, with γ-terpinene chemotype, is primarily dominated by oxygenated monoterpenes, such as γ-terpinene, p-cymene, and thymol, which was consistent with previous studies [48,49,50]. Based on their chemical compositions, *T. ammi* (Ethiopian variety) was characterized by a high level of γ-terpinene (γ-terpinene chemotype), whereas *T. ammi* from India was dominated by a high concentration of thymol (thymol-chemotype). Despite their difference in chemotypes, there was no significant difference in acaricidal activity of *T. ammi* originating from Ethiopia and India, demonstrating that thymol is the primary compound responsible for the acaricidal activity against mites. Studies have also demonstrated the high toxicity of *T. ammi* oil against other serious pests such as Dermanyssus gallinae (De Geer) [51], Aethina tumida (Murray) [50], Aedes aegypti (Linnaeus) [52], and Tuta absoluta (Meyrick) [53].

Thymol, a major component of *T. schimperi* Bisrat et al. [28], *T. ammi*, and commercialized miticide, exhibited strong acaricidal activity against *V. destructor*, consistent with previous studies [23,24,28,50,54,55]. Another monoterpenoid compound, γ-terpinene, occurring in high percentage in *T. ammi* showed low toxicity against *V. destructor*, with a 4 h LC_50_ = 1339 µg/mL (67 mg/L air volume). However, γ-terpinene displayed moderate toxicity against some pests, such as Aethina tumida [50], Tuta absoluta [53], and Hyalomma marginatum Koch, a common ectoparasite of passerine birds [56].

The toxicity of EOs to insects depends on various factors such as mode of action, concentration, mixing capacity and functional groups [57,58]. In our findings, a binary mixture of thymol and carvacrol, two major components with acaricidal activity, displayed strong synergistic effects against *V. destructor*, consistent with previous studies of a binary mixture against some pests [48,59,60]. The synergistic effect between the main components of EOs may be achieved through various mechanisms such as multi-target effects, pharmacokinetic or physicochemical effects, interactions with resistance mechanisms, or respective elimination or neutralization of adverse effects [48,61]. Thymol and carvacrol were also found to be stable under various environmental conditions, such as oxidation, hydrolysis, photolysis, and thermal exposure, making their mixture a promising alternative for controlling *V. destructor* [62]. However, antagonistic interaction was observed between the binary mixture of thymol:γ-terpinene (1:1) and the ternary mixture of thymol:carvacrol:γ-terpinene (1:1:1), which may be due to the low toxicity of γ-terpinene.

*E. globulus* and *R. officinalis* oils, dominated by 1,8-cineole, had low toxicity against *V. destructor* compared to previous studies [63,64]. However, the toxicity of 1,8-cineole, an inhibitor of AChE, varies depending on the target pests, as has been shown in different studies [27,65,66,67]. The inconsistency in results may be due to differences in experimental design, composition, and interaction of components.

According to our study, *T. schimperi* and *T. ammi* were both toxic to *V. destructor* compared with two honey bee species (*A. cerana* and *A. mellifera*) under laboratory conditions. Honey bees exposed to either oil or their major component through surface treatment showed low susceptibility (SR > 1) as shown in Table 5. These results align with reports showing that thyme oil had minimal toxicity to honey bees [62,68], and *T. ammi* powder did not affect bee workers’ activity, queen reproduction, brood, and adult development [69]. Furthermore, the plant-based formulation (Tinavar) has been reported to exhibit promising results in controlling varroosis while being safe to eggs, larvae, workers, and queens of honey bees [70]. In contrast, fluvalinate, a synthetic acaricide, was not selective towards *V. destructor*, with a selectivity ratio of 0.8 against A. cerana and 0.9 against *A. mellifera*. Our findings are consistent with the study of Gashout and Guzmán-Novoa. [24] that reported a selectivity ratio of 0.3 for fluvalinate against *A. mellifera* adults and larval. The reduced selectivity (SR < 1) in our study could be attributed to the development of resistance by *V. destructor*.

The selectivity of honey bees, on *T. schimperi*, *T. ammi* EOs, and their major components (thymol, carvacrol, and γ-terpinene) was found to be approximately 1.5 to 5 times higher towards the native bee, A. cerana, compared to *A. mellifera*. This difference could be attributed to the considerably lower average body mass of A. cerana (73.95 mg) in contrast to *A. mellifera* (99.45 mg) [71] that may affect the sensitivity toward cyano-neonicotinoid. Our finding is consistent with the study that demonstrated body weight was related to the toxicity of permethrin and methomyl in which the larger Melipona beecheii were less susceptible to both compounds compared with the smaller Trigona nigra [72]. Research has also indicated that *A. mellifera* of the same age tend to be more susceptible to pesticides (couphamos and fluvalinate) when they have smaller body weights maturing at 35 °C [73]. However, it contradicts the conclusion that toxicity is associated with chemical structure rather than body mass, as stated by Yue et al. [71]. The report by da Silva et al. [43], also highlighted the lower toxicity of EOs against Trigona hyalinata compared to *A. mellifera*, despite the latter having a larger body weight. The authors contend that factors such as fat deposit levels, hemolymph pH, and the number of detoxifying genes in cytochrome P450 play a more significant role in determining toxicity, rather than simply relying on body weight.

On topical exposure to *A. mellifera* to EOs, *T. ammi* was less toxic (LD_50_ = 14.7 µg/bee) than *T. schimperi* (LD_50_ = 7.9 µg/bee), with thymol being moderately toxic (LD_50_ = 4.6 µg/bee). However, thymol is of low or mild toxicity to honey bees with its string acaricidal properties against *V. destructor* [24]. Additionally, carvacrol showed promising results as a *V. destructor* mortality agent [27] and less toxicity to honey bees with minimal mortality being reported even at high concentrations (0.5%) [26]. This may be due to the low penetration rate of carvacrol in the cuticle of the bee, which is proportional to their lipophilicity [43] and/or rapid evaporation of the essence [68].

Sublethal effects evaluation is as important as lethal evaluation since honey bees are exposed to lower doses in realistic field conditions. Studies have shown that pesticides could have adverse effects on bee learning and memory following acute or chronic exposure [74,75]. For example, synthetic acaricides, fluvalinate, and coumaphos have big impacts on honey bee learning and memory at high doses [76]. However, in our study using PER assay, no reduction in olfactory learning and memory was observed in honey bees exposed to either *T. schimperi* EO or *T. ammi* EO or major constituents through topical application of sublethal doses (LD_10_, LD_20_) after 4 h. Even amitraz, a positive control in this study, did not affect learning and memory at a lower dose (LD_10_ 0.4 µg/bee). Although reports suggest that miticides have no effect on bee learning and memory, the toxicity of the pesticide adjuvant might be a factor [74]. Based on the goal of finding effective and natural alternatives to conventional acaricides, the EOs, major components, and mixtures of major compounds identified in this study have the potential to serve as alternatives for controlling *Varroa* mites in honey bee colonies.

## 5. Conclusions

Our research aimed to discover plant-based solutions that are both natural and eco-friendly, for controlling *Varroa* mites. These mites can have negative consequences on honey bees, leading to economic and ecological challenges. Through our study, we found two EOs, *T. ammi* and *T. schimperi*, and their components and a 1:1 mixture of thymol and carvacrol exhibited higher toxicity against ectoparasites mites that harm bees, while being safer for two species of honey bees. Additionally, these plant products are easily degradable, providing a practical and sustainable solution. At the end, we propose that by combining thymol and carvacrol, we could achieve better results in controlling *V. destructor* through an integrated pest management (IPM) approach. This method emphasizes the use of cultural and mechanical practices to control mites before resorting to chemical methods, whether mild or strong. However, further field studies are required to assess the impact of the thymol and carvacrol mixture on honey bees.

## Figures and Tables

**Table 1:** Percentage yields of essential oils (EOs) obtained from *Eucalyptus globulus, Rosmarinus officinalis,* and *Trachyspermum ammi*.

Plant Name	Yield (%) (*v*/*w*)	Odor	Color
*Eucalyptus globulus* (leaf)	1.10	strong pungent	coloress
*Rosmarinus officinalis* (leaf)	0.83	intense spicy aroma.	pale yellow
*Trachyspermum ammi* (seed)	3.7	Aromatic odor	Pale yellow
*Trachyspermum ammi* ^a^ (seed)	4.1	Aromatic odor	Pale yellow

Note: All the plant materials were collected from Ethiopia except *Trachyspermum ammi*. ^a^ Indian variety.

**Table 2:** Main components (%) detected by GC-MS in EOs.

No	Compounds ^a^	RI ^b^	Ri ^c^	*E. globulus*	*R. officinalis*	*T. ammi* ^d^	*T. ammi* ^e^
1	α-Pinene	931.7	936.1	15.19	3.84	0.58	0.04
2	β-Pinene	973.5	977.7	1.84	2.42	4.56	0.78
3	p-Cymene	1027.3	1024.3			27.92	17.72
4	1,8- Cineole	1033.1	1031.8	56.72	29.29		
5	γ-Terpinene	1061.2	1059.7	1.88		32.72	17.02
6	Camphor	1140.6	1143.4		16.08		
7	Isoborneol	1156.9	1158.2		7.32		
8	α-Terpineol	1190.4	1187.7	7.28		0.28	0.17
9	Carvestrene	1195.2			8.58		
10	Thymol	1292.5	1290.1		0.94	24.36	59.40
11	Carvacrol	1301.4	1300.4			0.51	0.12
12	Cis-Caryophyllene	1404.0	1406.5		6.69		

Note: ^a^ Compounds listed in order of elution; ^b^ RI and ^c^ RI are the Kovats retention indices determined relative to a series of *n*-alkanes (C9–C29) on a non-polar (HP5-MS type column) capillary column, respectively, under conditions listed in the Materials and Methods section; constituents of the EOs were identified by comparing their Kovats retention indices(RIs) with those reported in the literature [40] and their mass spectra with those listed in the Wiley mass spectral library. ^d^ Ethiopian variety, ^e^ Indian variety.

**Table 3:** Lethal concentration (LC_50_ (µg/mL), 95% confidence limits (CI) of EOs, their major constituents, and mixtures of major constituents against *V. destructor* estimated 4 h after exposure.

Treatments	Probit Analysis					
	N	LC_50_ (95% CL) (µg/mL)	Slope ± SE	Intercept	χ^2^	df
*Eucalyptus globulus*	75	4341.0 (2218.0–11,833.6)	3.9 ± 0.8		4.5	13
*Rosmarinus officinalis*	75	2577.0 (2013.0–3284.0)	4.5 ± 0.9	20.3	47.8	13
*Trachyspermum ammi* ^a^	75	98.0 (84.0–133.6)	2.7 ± 0.5	0.5	10.4	13
*Trachyspermum ammi* ^b^	75	81.0 (60.2–108.7)	3.2 ± 0.6	−1.1	5.6	13
Thymol	75	71.0 (52.5–95.8)	3.0 ± 0.6	−0.5	10.9	13
Carvacrol	75	106.0 (76.5–137.4)	3.9 ± 0.9	−2.9	5.5	13
Γ-Terpinene	75	1339.0 (848.0–3032.2)	1.6 ± 0.4	−0.1	5.1	13
p-cymene	75	>5000.0 (-)	-	-	-	-
1,8-Cineole	75	>10,000.0 (-)	-	-	-	-
Thymol:carvacrol	75	42.0 (35.2–47.9)	10.4 ± 2.9	11.8	4.1	13
Thymol:γ-terpinene	75	146.0 (112.7–222.8)	7.8 ± 2.2	−12.1	2.3	13
Thymol:carvacrol:γ-terpinene	75	113.0 (80.2–164.7)	1.5 ± 0.3	1.1	7.6	13
Fluvalinate	75	143.0 (51.6–1576)	0.8 ± 0.2	1.8	85.4	13

^a^ Ethiopian variety, ^b^ Indian variety.

**Table 4:** Lethal contact dose, LD_50_ (µg/bee) for *T. schimperi* and *T. ammi* EOs and their main constituents (thymol, carvacrol, and γ-terpinene) to *A. mellifera*.

Samples	Probit Analysis					
	N	LD_50_ (95% CL) µg/bee	Slope ± SE	χ^2^	Intercept	df
*Thymus schimperi*	180	7.9 (3.1–14.6)	0.5 ± 0.2	10.1	4.6	13
*Trachyspermum ammi*	180	14.7 (6.9–156.0)	0.8 ± 0.2	11.8	4.1	13
Thymol	180	4.6 (3.0–10.2)	1.4 ± 0.3	19.3	4.1	13
Carvacrol	180	9.9 (6.5–21.3)	1.5 ± 0.3	12.3	3.5	13
γ-Terpinene	180	86.5 (47.6–410.8)	0.8 ± 0.2	8.6	3.4	13
Amitraz	180	5.3 (2.1–47.7)	1.1 ± 0.2	39.2	4.2	13

**Table 5:** Selectivity ratio of *T. schimperi*, *T. ammi* EOs and their main constituents for *V. destructor* and honey bees.

Treatments	$\frac{{\mathrm{LC}}_{50}\;Ac}{{\mathrm{LC}}_{50}Vm}$	$\frac{{\mathrm{LC}}_{50}\;Am}{{\mathrm{LC}}_{50}\;Vm}$	$\frac{{\mathrm{LC}}_{50}\;Am}{{\mathrm{LC}}_{50}Ac}$
*Thymus schimperi*	27	91	3.4
*Trachyspermum ammi*	9.2	48	5.2
Carvacrol	28	38	1.4
Thymol	4.4	6.5	1.5
γ-terpinene	11.8	38	3.2
Fluvalinate	0.8	0.9	1.1

*Vm*—*Varroa* mite, *Ac*—*Apis cerena*, *Am*—*Apis mellifera*.

## Data Availability

The manuscript and supporting information contain all the data presented.

## Supplementary Materials

The following supporting information can be downloaded at: https://www.mdpi.com/article/10.3390/insects14090735/s1, Figure S1: Dose-response lines of essential oils than *E. globulus* (A), *R. officinalis* (B) and *T. ammi* (C) and their components 1, 8- cineole, thymol and γ-terpinene 4-h after of exposure; Figure S2: Dose-response lines of *T. ammi* and its main components (thymol, and γ-terpinene) to *A. mellifera* and A. cerana 4-h after treatment exposure; Figure S3: Dose-response lines of T. schimepri and its main components (thymol, and carvacrol) to *A. mellifera* and A. cerana 4-h after treatment exposure. Figure S4: Dose-response lines of the mortality of *T. schimperi* (A) and *T. ammi* (B) and their major components (carvacrol, thymol, γ-terpinene) to *A. mellifera* 4-h post topical exposure. Table S1: All Main components (%) detected by GC-MS in essential oils; Table S2: Estimated 4-h post exposure lethal concentration (LC50), 95% confidence limits (CL) *T. schimperi* and *T. ammi* EOs, their major components (thymol, carvacrol and γ-terpinene) and tau-fluvalinate against honey bees.
