# Supplementary material for: Acaricidal Toxicity of Four Essential Oils, Their Predominant Constituents, Their Mixtures against Varroa Mite, and Their Selectivity to Honey Bees (Apis cerana and A. mellifera)
Source: Insects. 2023 Aug 30;14(9):735. doi: 10.3390/insects14090735 (PMC10532382; doi:10.3390/insects14090735)
Supplement: Supplementary file 1 [file insects-14-00735-s001.zip › insects-2511680-supplementary.pdf]

Supporting Information

GC-MS analysis result of EOs

Table S1. All Main components (%) detected by GC-MS in essential oils

No	Compounds[a]	RI[b]	Ri[c]	*E. globulus*	R. *officinalis*	*T. schimperi*[d]	*T. ammi*[e]	*T. ammi*[f]
1	α-Thujene	926.3	927.8	0.18		0.92	0.79	0.051
2	α-Pinene	931.7	936.1	15.19	3.84	0.25	0.58	0.037
3	Camphene	947.8	950.3	0.41	2.89			
4	β-Pinene	973.5	977.7	1.84	2.42	0.07	4.56	0.78
5	1-Octen-3-ol	980.2	980.0			0.40		
6	3-Octanone	986.2	984.5			1.63		
7	β-Myrcene	991.8	989.2	2.38		0.92	1.07	0.225
8	3-Octanol	996.4	993.2			0.82		
9	α-Phellandrene	1002.4	1004.1	2.81		0.18	0.94	
10	3-Carene	1008.6	1011.3				0.12	
11	α-Terpinene	1015.8	1017.1			1.96	0.55	0.249
12	p-Cymene	1027.3	1024.3			14.20	27.92	17.72
13	Limonene	1027.3	1029.5					0.117
14	α-Sabinene	1029.7	-			0.88		
15	1,8- Cineole	1033.1	1031.8	56.72	29.29			
16	γ-Terpinene	1061.2	1059.7	1.88		8.52	32.72	17.02
17	(Z)-Sabinene hydrate	1065.7	1066.5			0.35	0.14	0.043
18	Cis-Linanool oxides	1072.1	1075.1	0.15				
19	α-Terpinolene	1087.3	1086.9			0.29		0.172
20	(E)-Sabinene hydrate	1096.0	1098.1				0.17	
21	Linalool	1100.4	1099.0		2.73	2.68	1.21	0.045
22	Undecane	1102.1	1100.0				0.12	
23	Camphor	1140.6	1143.4		16.08			
24	(Z)-Verbenol	1149.5	1144.4				0.13	
25	Isoborneol	1156.9	1158.2		7.32			
26	P-Mentha-1,5-dien-8-ol	1166.0	1166.6				1.21	
27	Terpinen-4-ol	1175.8	1177.1	1.06	2.48	1.16	0.85	0.374
28	α-Terpineol	1190.4	1187.7	7.28		0.74	0.28	0.168

29	Carvestrene	1195.2			8.58			
30	(E)-Dihydrocarvone	1203.5	1201.4			0.15		
31	(Z)-Dihydrocarvone	1217.9	-			0.10		
32	Thymol methyl ether	1243.3	1234.3			0.15		
33	Geraniol	1252.6	1254.9	0.64				
34	(Z)-2,3- Epoxydecane	1264.2	-				0.15	
35	Trans-Anethole	1280.7	1285.2		0.43			
36	Bornyl acetate	1282.4	1285.2		1.92			
37	Thymol	1292.5	1290.1		0.94	23.03	24.36	59.402
36	Carvacrol	1301.4	1300.4			34.84	0.51	0.123
39	α-Cubebene	1349.5	1351.4		0.95			
40	Thymol acetate	1355.3	1356.4			0.55	0.20	
41	Eugenol	1356.5	1357.8					0.57
42	Carvacryl acetate	1373.2	1373.1			0.75	0.10	0.26
43	Geranyl acetate	1380.9	1379.9	0.62				
44	Cis-Caryophyllene	1404.0	1406.5		6.69			
45	(E)-Caryophyllene	1422.3	1420.1			0.49		
46	β-Humulene	1439.9	1442.5		1.58			
47	Aromandendrene	1440.4	1440.6	3.13	1.24			
48	γ-Muurolene	1471.4	1476.2		1.43			
49	(Z)-β-Farnesene	1486.7	-			0.04		
50	α-Muurolene	1495.2	1498.3		0.51			
51	α-Bisabolene	1503.6	1508.4		0.46			
52	β-Bisabolene	1510.9	1508.4			0.04		
53	β-Sesquiphellandrene	1524.7	1523.5			0.18		
54	Caryophyllene oxide	1582.7	1580.6		2.63	0.38		
55	γ-Eudesmol	1632.1	1630.9	0.34				
56	Epi-α-Cadinol	1639.5	1637.8	0.26				
57	α-Muurolol	1645.6	1642.9	1.28				
	Total			96.2	95.4	96.7	98.7	96.8

Note: [a] Compounds listed in order of elution; [b] RI and [c] RI are the Kovats retention indices determined relative to a series of *n*-alkanes (C9–C29) on a non-polar (HP5-MS type column) capillary column, respectively, under conditions listed in the Materials and Methods section; constituents of the essential oils were identified by comparing their Kovats retention indices(RIs) with those reported in the literature (Babushok, 2011) and their mass spectra with those listed in Wiley mass spectral library. [d]Bisrat et al., [28], [e]Ethiopian variety, [f]Indian variety

Dose Response Curve of the EOs and Their Major Components on *Varroa destructor*

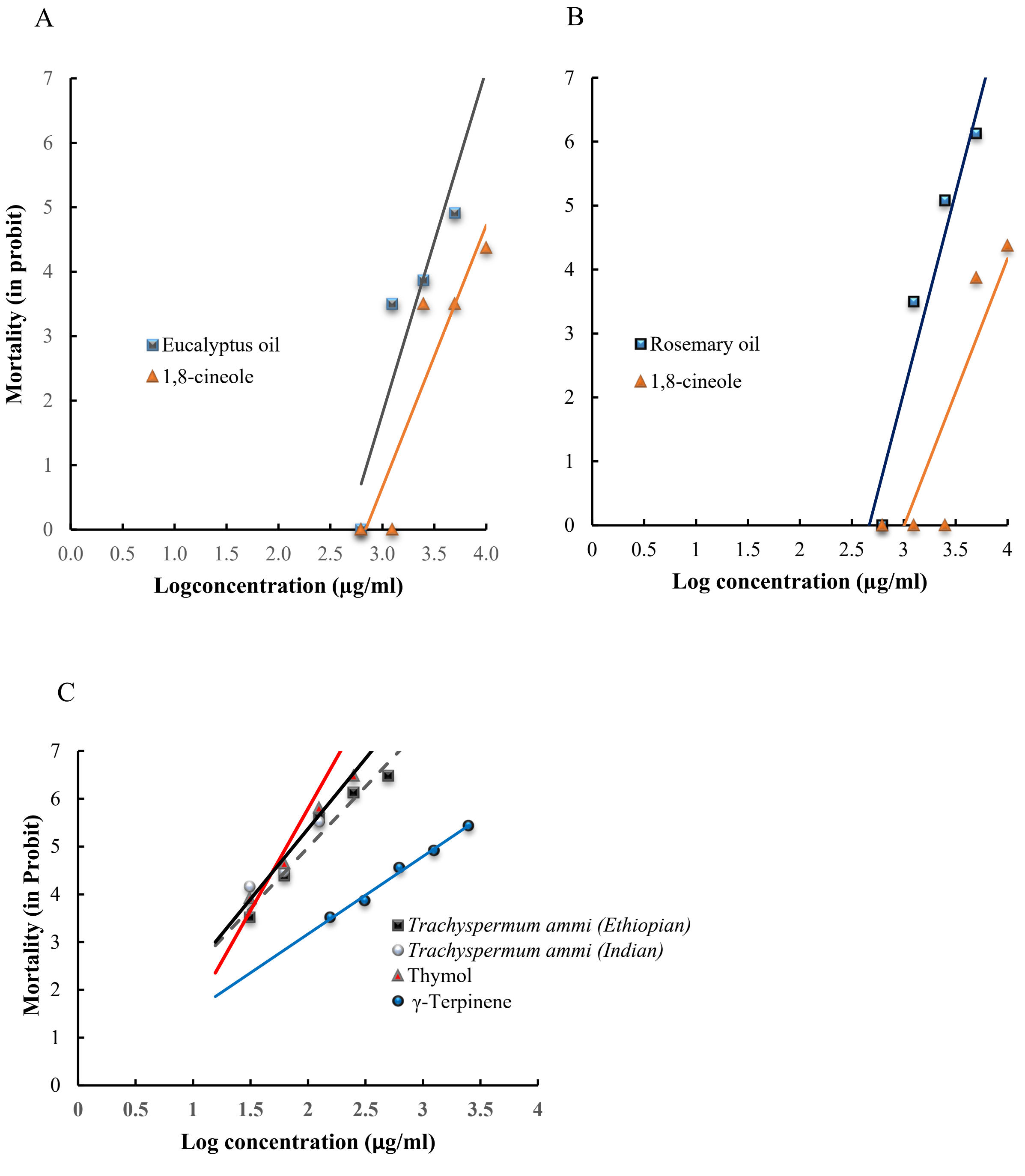

Figure S1.Dose-response lines of essential oils than *E. globulus* (A), *R. officinalis* (B) and *T. ammi* (C) and their components 1, 8- cineole, thymol and γ-terpinene 4-h after of exposure.

Surface Treatment Dose Response Curve of *T. ammi* and Its Major Components on Honey bees

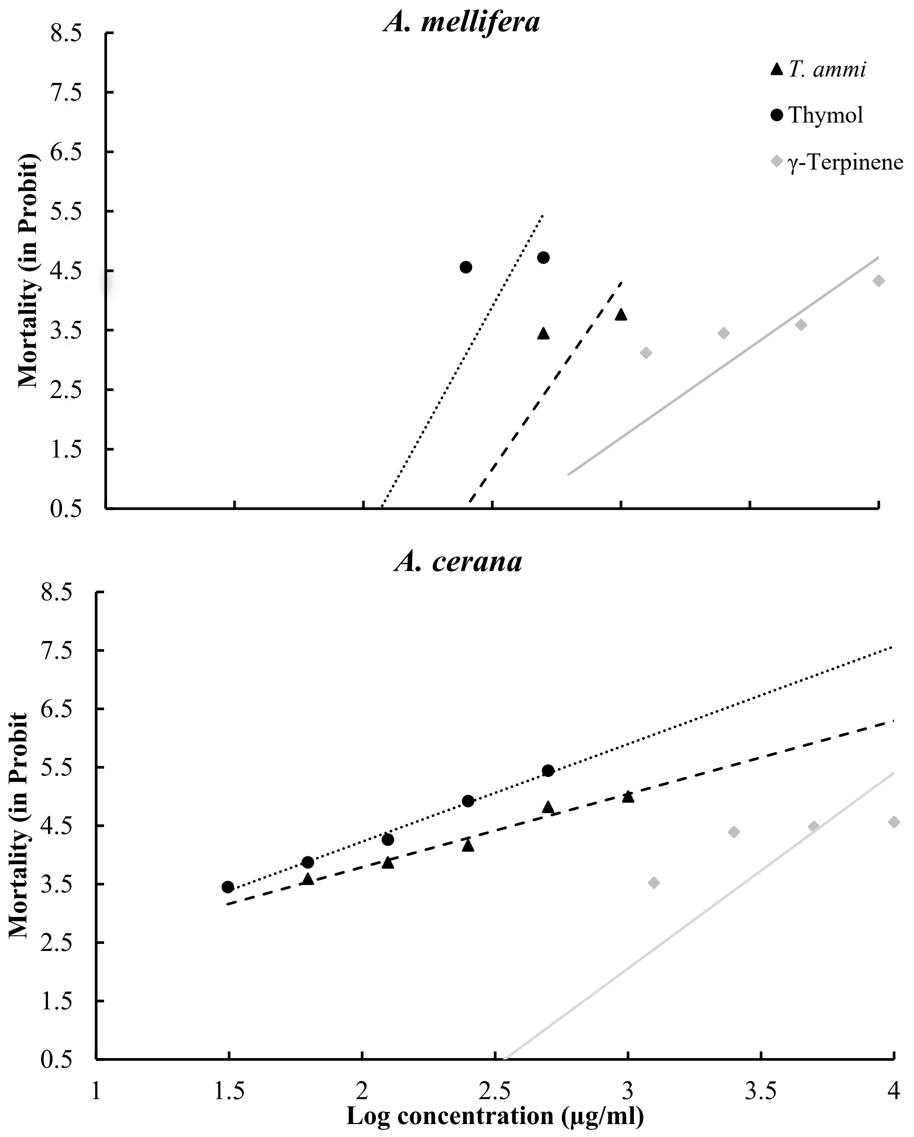

Figure S2. Dose-response lines of *T. ammi* and its main components (thymol, and γ-terpinene) to *A. mellifera* and *A. cerana* 4-h after treatment exposure.

Surface treatment Dose response curve of *T. schimepri* and Its Major Components on Honey bees

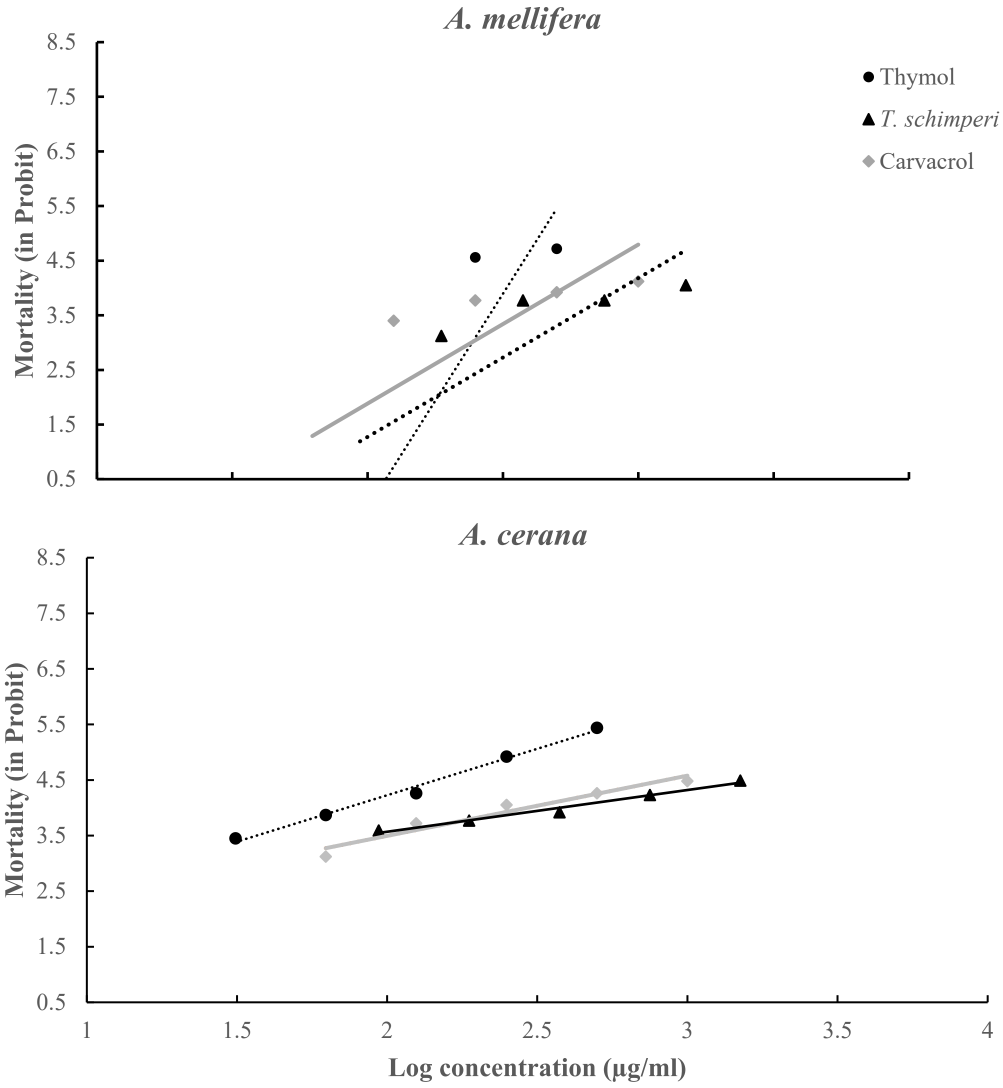

Figure S3. Dose-response lines of *T. schimepri* and its main components (thymol, and carvacrol) to *A. mellifera* and *A. cerana* 4-h after treatment exposure

Estimated LC_{50} of *T. schimperi*, *T. ammi* and Their Major Components on Honey bees

Table S2. Estimated 4-h post exposure lethal concentration (LC_{50}), 95% confidence limits (CL) *T. schimperi* and *T. ammi* EOs, their major components (thymol, carvacrol and γ-terpinene) and tau-fluvalinate against honey bees.

Test samples	Species	Probit Analysis						
		**N**	LC_{50}	95% CL	Slope ± SE	Intercept	χ^2	df
T. schimperi	*A. mellifera*	180	9971	2868-16,300	1.1 ± 0.4	0.7	6.9	13
	A. cerana	150	2946	1017- 4131	0.8 ± 0.2	-2.2	2.9	13
T. ammi	*A. mellifera*	180	4700	1553 -5911	1.9 ± 0.9	-2.0	10.3	13
	A. cerana	150	943	572.9-2267	1.2 ± 0.3	2.7	14.6	13
Thymol	*A. mellifera*	180	460.9	259 –1698	2.9 ± 0.6	-2.6	71.4	13
	A. cerana	150	315	229 – 506	1.8 ± 0.3	0.8	12.9	13
Carvacrol	*A. mellifera*	180	4107	1592-	3.7 ± 0.4	0.4	3.8	13
	A. cerana	150	2994	1135-	4.0 ± 0.3	1.6	3.6	13
γ-Terpinene	*A. mellifera*	180	51129	13655-78463	1.2 ± 0.4	0.8	22.7	13
	A. cerana	150	15826	6033-22180	1.2 ± 0.3	0.2	35.4	13
Fluvalinate	*A. mellifera*	180	135	63-355	0.8 ± 0.1	3.3	4.5	13
	A. cerana	150	120	99-266	0.8 ± 0.1	3.4	4.8	13

Topical Exposure Dose Response Curve of *T. schimperi* and *T. ammi* and Their Major Components on *A. mellifera*

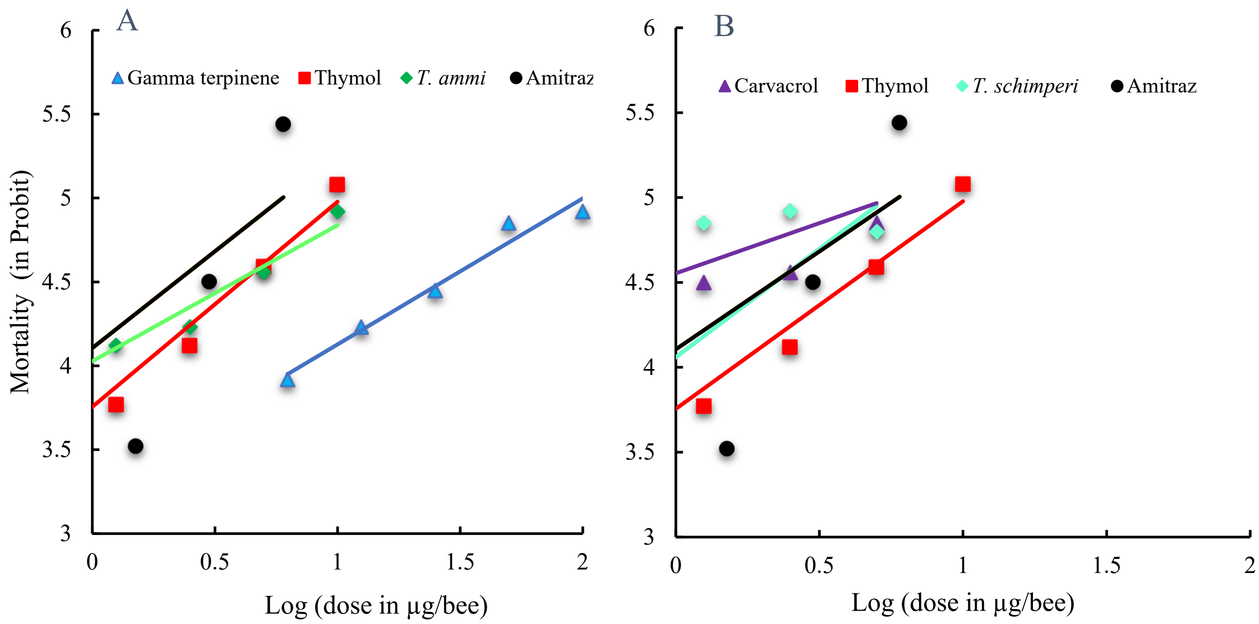

Figure S4. Dose-response lines of the mortality of *T. ammi* (A) and *T. schimperi* (B) and their major components (carvacrol, thymol, ɣ-terpinene) to *A. mellifera* 4-h post topical exposure
